# High number of hypoglycaemic episodes identified by CGM among home-dwelling older people with diabetes: an observational study in Norway

**DOI:** 10.1186/s12902-023-01472-6

**Published:** 2023-10-10

**Authors:** Mari Fløde, Monica Hermann, Anne Haugstvedt, Eirik Søfteland, Jannicke Igland, Anders Åsberg, Trond Geir Jenssen, Marit Graue

**Affiliations:** 1 Department of Health and Caring Sciences, Western Norway University of Applied Sciences, Bergen, Norway; 2 Department of Medicine, Haukeland University Hospital, Bergen, Norway; 3 Department of Clinical Science, Faculty of Medicine, University of Bergen, Bergen, Norway; 4 Department of Global Public Health and Primary Care, University of Bergen, Bergen, Norway; 5 Department of Transplantation Medicine, Oslo University Hospital, Oslo, Norway; 6 Department of Pharmacy, Section for Pharmacology and Pharmaceutical Biosciences, University of Oslo, Oslo, Norway; 7 Institute of Clinical Medicine, University of Oslo, Oslo, Norway

**Keywords:** Diabetes, Older people, Hypoglycaemia, Home care, Clinical pharmacology, Drug safety

## Abstract

**Background:**

A scoping review from 2021 identified a lack of studies on the incidence, prevention and management of hypoglycaemia in home-dwelling older people with diabetes. The aim of this study was to investigate the frequency and duration of hypoglycaemic episodes measured by continuous glucose monitoring (CGM) in older people with diabetes who received home care and who were treated with glucose-lowering medications, and to compare the frequency and duration of hypoglycaemic episodes between subgroups of the study population according to demographic and clinical variables.

**Methods:**

This was an observational study investigating the occurrence of hypoglycaemia in people with diabetes aged ≥ 65 years. Data were collected using blinded continuous glucose monitoring (CGM, iPro2) for 5 consecutive days. Frequency and duration of hypoglycaemic episodes were assessed using a sensor glucose cut-off value of 3.9 mmol/L. A blood sample for measurement of HbA1c and creatinine-based eGFR (CKD-EPI) was obtained during the monitoring period. Demographic and clinical data were collected from electronic patient records.

**Results:**

Fifty-six individuals were enrolled (median age 82 years and 52% were men). Of the 36 participants who were treated with insulin, 33% had at least one hypoglycaemic episode during the five-day period. Among 18 participants who neither used insulin nor sulfonylurea, but other glucose-lowering medications, 44% had at least one hypoglycaemicepisode. Of those with hypoglycaemic episodes, 86% lived alone. The median duration of the hypoglycaemia was 1 h and 25 min, ranging from 15 min to 8 h and 50 min.

**Conclusion:**

This study identified an unacceptably high number of unknown hypoglycaemic episodes among older home-dwelling people with diabetes receiving home care, even among those not using insulin or sulfonylurea. The study provides essential knowledge that can serve as a foundation to improve the treatment and care for this vulnerable patient group. The routines for glucose monitoring and other prevention tasks need to be considered more comprehensively, also, among those treated with glucose-lowering medications other than insulin.

**Supplementary Information:**

The online version contains supplementary material available at 10.1186/s12902-023-01472-6.

## Background

Older home-dwelling people with diabetes have an increased risk of hypoglycaemia due to frailty, impaired cognitive function, poor nutritional status, and polypharmacy [1, 2]. Moreover, impaired hormonal counter regulation with lack of autonomic warning symptoms in addition to increased drug response due to age-related changes in drug metabolism and excretion, also increase the risk of hypoglycaemia [3–6]. Hypoglycaemia in older people with diabetes is associated with adverse outcomes and contributes to an increased risk of dangerous and life-threatening cardiac complications [7], risk of falls and fractures, cognitive decline, and acceleration of dementia [8, 9], and, hypoglycaemic episodes double the overall mortality in older people [10]. We know that recurrent episodes of non-severe hypoglycaemic epiodes are also associated with physical and cognitive decline [2]. Finally, in older persons the threshold for autonomic and cognitive symptoms is at a lower glucose level compared with younger adults, which increases the risk of more severe hypoglycaemic episodes [10].

A previous study in Norway showed that approximately 30% of people > 80 years of age received home care services, and among these, about 24% had diabetes [11]. For older people with diabetes, home care services often include help with administration of medication, glucose monitoring and follow-up of diabetes-associated complications. A scoping review from 2021 identified, however, a lack of studies on the incidence, prevention and management of hypoglycaemia in home-dwelling older people with diabetes [1]. Most studies were based on data from ambulance records or emergency hospital visits, and the frequency and duration of hypoglycaemia handled by the persons themselves, a home care nurse or the individuals’ next-of-kin have therefore not been well reported [1]. Given the limited knowledge and the significant consequences of hypoglycaemia in older people with diabetes, studies using continuous glucose monitoring (CGM) are warranted to provide more knowledge on this important topic. Thus, the aim of this study was 1) to investigate the frequency and duration of hypoglycaemic episodes measured by continuous glucose monitoring (CGM) in older people ≥ 65 years with diabetes who received home care and were treated with glucose-lowering medications, and 2) to compare the frequency of hypoglycaemia between subgroups of the study population according to demographic and clinical variables.

## Methods

### Study design

A five -day observational study was conducted among people ≥ 65 years with diabetes who received home care services in two home care zones in one municipality in Western Norway.

### Setting and study sample

The participants were recruited between January 2020 and December 2021 from the total population of individuals with diabetes. These two home care zones comprise approximately 39% of the total patient population in the municipality’s home care services. The patients in home care receive follow-up in their diabetes management from their general practitioner in primary health care. Those with type 1 diabetes generally also receive diabetes follow-up in the specialist health service. All eligible individuals who fulfilled the inclusion criteria were invited to participate in the study. The inclusion criteria were age ≥ 65 years, diagnosis of diabetes, treated with either insulin or any other glucose-lowering medication, adequate cognitive functioning, being able to communicate in Norwegian, and able to give written consent to participate. We excluded those with certain diseases that affect glucose regulation (malfunction of the adrenal cortex, malfunction of the pituitary gland, liver failure or surgically removed ventricle), those who already used CGM, and those with a severe somatic or psychiatric comorbidity (e.g., known end-stage renal disease, severe heart failure, severe cancer, severe depression or bipolar disorder, psychosis). We received information on age, diabetes diagnosis and medication from the electronic records of everyone with diabetes who received home care in the municipality. We subsequently reviewed their medical records to weed out those who did not receive blood glucose-lowering treatment or met other exclusion criteria. The rest of the participants were considered as eligible for the study. The study nurses then asked all with adequate cognitive function if they wanted to participate and included those accepting consecutively. The study nurses’ evaluation of cognitive function was done without any structured assessment tool. The evaluation was based on the nurse’s perception of the patient's cognitive function and whether they were able to give written informed consent. In addition to recruiting participants from the list gained from electronic patient records at study start, the study nurses continuously recruited new participants who entered the home care service upon the same inclusion and exclusion criteria until the end of the recruitment period. Unfortunately, because of logistic problems we could not expand the study period further after December 30th, 2021.

### Data collection and variable

We used a blinded continuous interstitial glucose monitoring system (Ipro2 CGM using Enlite glucose sensors; Medtronic MiniMed, Northridge, CA, USA) [12]. These sensors have an overall mean absolute relative difference (MARD) of 13.9%, sensitivity of 79.5%, and positive predictive value of 83.8% for hypoglycaemia [13]. MARD is the average of the absolute error between all CGM values and matched reference values. A small percentage indicates that the CGM readings are close to the reference glucose value, whereas a larger MARD percentage indicates greater discrepancies between the CGM and reference glucose values [14]. The less the MARD is, the closer are the CGM readings to the comparison values measured by the home care provider or the participants themselves [15]. No prospective clinical studies have evaluated the possible added clinical benefits of low MARD values [12, 14]. The Ipro2 requires capillary blood glucose samples for calibration. Capillary blood glucose samples for calibration were taken either by the participants themselves or the home care providers three times daily during the 5 days. For almost all participants capillary blood samples were part of routine follow-up prior to the study and the patient/home care provider was therefore familiar with the procedure. Routine blood glucose measurements varied in relation to whether they were on insulin or other blood glucose-lowering treatment, but for those on insulin it was mainly one recording in the morning and one in the evening. Those without insulin measured blood glucose once a week or every 14 days. During the study, blood glucose was measured three times daily for all participants. The level of home care varied from one to four visits per day. The participants’ daily life routines and treatment of diabetes were as usual during the monitoring period. The participants’ glycosylated haemoglobin (HbA1c) and serum creatinine levels were measured once during the study period and estimated glomerular filtration rate (eGFR) was determined using the CKD-EPI creatinine Equation (2021) [16].

Data on the frequency and duration of hypoglycaemic episodes were collected from the CGMs. Hypoglycaemia was defined as glucose values < 3.9 mmol/l for ≥ 15 min and recovery were obtained when the glucose value was continuously at or above 3.9 mmol/l for ≥ 20 min [17]. Nocturnal hypoglycaemia was defined as any hypoglycaemic episodes occurring between 22.00 h and 06.00 h. We collected data on recent hypoglycaemic episodes and also hypoglycaemia awareness, using The McKellar Risk Assessment Tool [18], which contains questions about issues associated with increased risk factors for hypoglycaemia, recognizing symptoms, mental state, treatment goals, treatment (insulin/sulfonylurea), kidney or liver disease, recent episode of hypoglycaemia, other medications and nutrition [18]. The definition of recent was not specified in the question.

Data on nutritional status were assessed by the self-reported instrument Mini Nutritional Assessment (MNA) during the five-day study period The goal of this assessment is to determine who is at risk of malnutrition, and hence to permit early nutritional intervention. The MNA includes anthropometric assessments (weight, height, and weight loss), general assessments (lifestyle, medication and mobility), dietary assessments (number of meals, food and fluid intake, autonomy of eating), and self-assessments (self-perception of health and nutrition). Based on scores from the assessments, the instrument classifies the person as well-nourished, undernourished, or at risk of malnutrition [19]. We chose to use the MNA to evaluate nutritional status because it is a well-known instrument used in a variety of settings. According to Bauer et al. [19], it is well suited for use as a nutrition screening tool also in elderly populations [19].

Demographic (sex, living conditions) and clinical data (type of diabetes, type of glucose-lowering drugs, age, weight, height) were collected from the electronic patient records. We used a cut-off BMI on < 24 kg/m^2^, since the optimal range of BMI for elderly people is 24–29 kg/m^2^ [20]. From the electronic patients records we also collected information about duration of medical treatment, co-medications and earlier severe (level 2) and non-severe (level 1) hypoglycaemic episodes.

### Statistics

Sociodemographic and clinical characteristics of the study population are reported as medians and range (min–max) and frequencies and percentages for categorical variables. Results are stratified for the use of insulin. The subgroup of participants with at least one hypoglycaemic episode is described in terms of number of hypoglycaemic episodes, number of episodes in different glucose intervals, median (range) total duration of hypoglycaemia, glucose variability measured as the coefficient of variation, median (range) HbA1c (mmol/mol [%]), eGFR (mL/min/1.73 m^2^), BMI (kg/m^2^) and weight (kg). Median (range) was reported due to the small sample size and skewed data. Glucose variability was measured using CV and range (min–max). The magnitude of glycaemic excursions, and the number, timepoint and duration of hypoglycaemic episodes are presented graphically.

We have performed descriptive statistics. Because of the small sample size, we did not have sufficient statistical power to test for statistically significant differences between insulin users and non-users. All analyses were performed in Stata version 17. The figure is made in R.

## Results

### Characteristics

The demographic and clinical characteristics of the 56 participants are summarized in Table 1. Thirty-six of the participants (64%) used insulin alone (*n* = 13) or in combination with other blood glucose-lowering medication (*n* = 23) (Table 2). Among the 23 (41%) persons who used insulin combined with other blood glucose-lowering medications, 13 (57%) used a dipeptidyl peptidase IV (DPP-4) inhibitor, 9 (39%) used metformin, 3 (13%) used a Glucagon-Like Peptide-1(GLP-1) receptor agonist, and 3 (13%) used a Sodium-Glucose Transport Protein 2 (SGLT2) inhibitor. Regarding the 36 insulin users with or without other glucose-lowering medications, 30 (83%) used two insulin treatment modalities in combination. Of the 20 persons who did not use insulin, two were treated with sulfonylurea, four were treated with metformin in monotherapy, and the rest used different combinations of metformin, SGLT2 inhibitors, and DPP-4 inhibitors. The median number of medications per participant was 10, ranging from 5 to 24.

**Table 1:** The characteristics of the 56 participants (≥ 65 years) with diabetes who received home-care services

Variables	Total sample (*n* = 56)	Insulin (*n* = 36)	Non-insulin (*n* = 20)
Age (years), median (range)	82 (65–99)	80 (65–94)	84 (70–99)
Gender			
Men, *n* (%)	29 (52)	15 (42)	14 (70)
Women, *n* (%)	27 (48)	21 (58)	6 (30)
Living alone, *n* (%)^a^	40 (71)	26 (72)	15 (75)
Type of diabetes			
Type 1, *n* (%)	7 (12)	7 (19)	0 (0)
Type 2, *n* (%)	49 (88)	29 (81)	20 (100)
HbA1c (mmol/mol), median (range)^a^	57 (34–108)	66 (39–108)	48 (34–53)
HbA1c (%), median (range)^a^	7.4 (5.3–12.0)	8.2 (5.7–12.0)	6.5 (5.3–7.0)
eGFR (ml/min/1.73 m^2^), median (range)^a^	69 (9–123)	63 (9–123)	86 (30–114)
Hb (g/dl), median (range)^a^	12.8 (9.7–16.3)	12.7 (9.7–16.3)	12.9 (10.1–14.4)
Weight (kg), median (range)^a^	78 (45–131)	78 (62–131)	78 (45–113)
Body mass index (kg/m^2^), median (range)^a^	27 (19–41)	28 (21–39)	25 (19–41)

*eGFR* estimated glomerular filtration rate

^a^Missing data: HbA1c, Hb, eGFR, *n* = 7; weight, *n* = 1; body mass index, *n* = 3

**Table 2:** Type of treatment for the 56 participants with diabetes (≥ 65 years) receiving home care

Type of treatment	Number (%)
Insulin (*n* = 36)	
Only rapid-acting insulin	0 (0)
Only slow-acting insulin	2 (6)
Only rapid-acting and slow-acting insulin	11 (30)
Only rapid-acting insulin and other glucose-lowering medication	1 (3)
Only slow-acting insulin and other glucose-lowering medication	3 (8)
Rapid-acting insulin, slow-acting insulin, and other glucose-lowering medication	19 (53)
Glucose-lowering medication other than insulin (*n* = 20)	
Metformin	17 (85)
Sulfonylurea	1 (5)
Glipizide	1 (5)
GLP-1 analogue	1 (5)
DPP-4-inhibitor	10 (50)
SGLT2-inhibitor	3 (15)

### The frequency and duration of hypoglycaemia measured by CGM

**Fig. 1:**
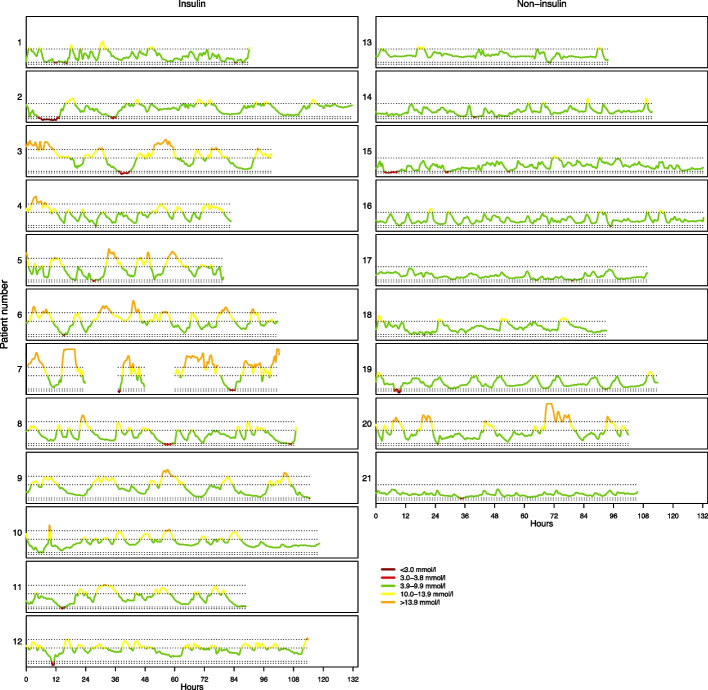
The glucose curves for the 21 participants with at least one hypoglycaemic episode, divided into inulin users and non-insulin users

During the study period, 21 (38%) of the 56 participants had one or more hypoglycaemic episodes, 12 (33%) of the insulin users and 9 (45%) of the non-insulin users (Table 3). The glucose curves for the 21 participants with at least one hypoglycaemic episode are shown in Fig. 1. Seventeen (46%) of the 37 hypoglycaemic episodes were defined as nocturnal hypoglycaemia. The rest of the episodes occurred at different times during the day, and only four episodes were identified by routine blood glucose measurements. The duration of the hypoglycaemic episodes in glucose level 1 (3.0–3.8 mmol/L) varied from 15 min to 8 h and 17 min (median 1 h and 15 min) and in level 2 (< 3.0 mmol/L) from 30 min to 6 h and 25 min (median 1 h and 30 min).

**Table 3:** Number of hypoglycaemic episodes, glucose level, duration of episodes, HbA1c level, eGFR level, weight, body mass index and glucose variability in participants with diabetes (≥ 65 years) receiving home care, with one or more hypoglycaemic episodes during the study period (*n* = 21)

	Total sample (*n* = 21)	Insulin (*n* = 12)	Non-insulin (*n* = 9)
Number of hypoglycaemic episodes, *n* (%)			
1	11 (52)	7 (58)	4 (44)
2–3	8 (38)	4 (33)	4 (44)
4–5	2 (10)	1 (8)	1 (11)
Glucose level assessed during hypoglycaemia, *n* (%)			
Level 1 3.0–3.8 mmol/l	16 (76)	9 (75)	7 (78)
Level 2 < 3.0 mmol/l	5 (24)	4 (33)	1 (11)
Duration of hypoglycaemia (h), median (range)	1.25 (0.15–8.50)	1.45 (0.15–8.50)	0.36 (0.25–6.0)
HbA1c (mmol/mol), median (range)^a^	52 (38–80)	58 (48–80)	42 (38–48)
HbA1c (%), median (range)^a^	6.9 (5.6–9.5)	7.5 (6.5–9.5)	6.0 (5.6–6.5)
eGFR (ml/min/1.73 m^2^), median (range)^a^	61 (22–110)	58 (22–101)	66 (40–110)
Weight (kg), median (range)^a^	75 (63–96)	72 (63–96)	77 (64–92)
Body mass index (kg/m^2^), median (range)^a^	24 (21–39)	28 (22–39)	24 (21–32)
Glucose variability (CV), median%(range)	28.8 (14.2; 39.4)	31.8 (20.3; 39.4)	24.2 (14.2; 39.1)

*eGFR* estimated glomerular filtration rate

^a^Missing data: HbA1c, *n* = 3; eGFR, *n* = 3; weight, *n* = 1; body mass index, *n* = 2

In this CGM-period median of 4.9 days, the median time below range (TBR) among those with one or more hypoglycaemic episodes was 2.2% (0.21–13.0) among those using insulin and 1.0% (0.4–6.2) among those not using insulin. The median time in range (TIR) among those with one or more hypoglycaemic episodes was 61.1% (18.0–88.6) among those using insulin and 95.3% (62.7–99.1) among those not using insulin. The median time above range (TAR) among those with one or more hypoglycaemic episodes was 38.7% (7.5–59.4) among those using insulin, and 3.6% (0.0–35.2) among those not using insulin.

### Frequency of hypoglycaemia according to the demographic and clinical characteristics

The median age among those who had a hypoglycaemic episode was 83 years (range 67–94 years), and 52% were men, and 86% of them lived alone compared to 71% of the total sample. HbA1c was lower among those with one or more hypoglycaemic episodes, than among those without hypoglycaemic episode (52 mmol/mol (38–80) vs 60 mmol/mol (34–108)) (Suppl. file 1). However, seven (33%) of the 21 participants with hypoglycaemia had HbA1c (≥ 53 mmol/mol (≥ 7.0%) (range 56–80 mmol/mol (7.3%–9.5%)). Hypoglycaemic episodes before the study period were not documented in the electronic patient records.When the study nurses asked, twenty (36%) of the 56 participants answered that they did not know the symptoms of hypoglycaemia or did not know when they had low blood glucose. Eight (40%) of these 20 participants had one or more hypoglycaemic episodes during the study.

Fifteen (27%) of the participants had BMI < 24 kg/m2, the median among these was 22.8 kg/m2 (range 18.9–23.9). Seven of those had one or more hypoglycaemic episodes and three used glucose-lowering medications other than insulin. According to the MNA assessment, 12 (21%) of all participants were at risk of malnutrition or were malnourished. The rest 44 (79%) participants had normal nutritional status. Two (22%) of the nine participants with hypoglycaemia and not on insulin were at risk of malnutrition. Among the 12 who used insulin and who underwent hypoglycaemic episodes, 2 (17%) were at risk of malnutrition.

We had eGFR data available on 49 participants (7 missing). Of the 14 (29%) participants with eGFR < 30 mL/min/1.73 m^2^ (stage G4 or G5 (moderate or severe kidney failure)), 36% had at least one hypoglycaemic episode. The eGFR ranged from 24 to 52 mL/min/1.73 m^2^ (data missing for two) among those with level 2 hypoglycaemia (< 3.0 mmol/L).

## Discussion

In this observational study, we found that one out of three older participants using insulin had at least one hypoglycaemic episode within the five-day study period. Surprisingly, almost half of the non-insulin group had at least one level 1 hypoglycaemic episode (3.0–3.8 mmol/L), and one participant had a level 2 episode (< 3.0 mmol/L). Only one of these participants was treated with sulfonylurea. The rest used metformin alone or in combination with DPP-4-inhibitors. Our findings are made in a population without any prior suspicion of hypoglycaemia, in contrast to a previous study, which also detected hypoglycaemia in almost half of the participants (adults ≥ 40) but where inclusion was based on a clinical suspicion of hypoglycaemia [21]. The hypoglycaemic episodes identified in our study occurred mostly at times other than those for routine blood glucose measurements carried out by home care services. Almost half of the episodes were defined as nocturnal hypoglycaemia, indicating that most of the episodes were not identified by the home care providers. Moreover, none of the participants reported these recorded episodes themselves and most of the hypoglycaemic episodes lasted for at least one hour. A recent study supported these results and concluded that nocturnal hypoglycaemia is very common and largely underdiagnosed in older people with insulin-treated type 2 diabetes. They also demonstrated that most of hypoglycaemic episodes in older people are asymptomatic, especially during the night [22].

A previous study showed that the average glucose among healthy nondiabetic persons over 60 years old is 5.8 mmol/L [23]. The median time spent with glucose levels < 3.9 mmol/L was 1.1% (15 min/d) [23].

We consider that the results from our study add important information to this field of research. The participants had a median age of 82 years, the oldest participant was 99 years old, and all of them received regular home care services. Therefore, it is worrying that we found such a high number of hypoglycaemic episodes in this vulnerable group of home care users, particular in the light of previous research showing that recurrent hypoglycaemic episodes may be associated with impaired cognitive function and development of dementia [2]. The brain is highly dependent on glucose for its metabolism and is particularly vulnerable to hypoglycaemia especially in older people. After each hypoglycaemic episode major cognitive changes may occur leading to post-hypoglycaemic encephalopathy [2]. In addition to dementia, a meta-analysis demonstrated significant associations between hypoglycaemia and death, macrovascular and microvascular complications, cardiovascular death, and falls and fractures [7]. There may, however, be some reasonable explanations for the high number of hypoglycaemic episodes identified in this group of older people with diabetes. More than 70% of the participants had reduced kidney function, and even a mild reduction in kidney function is shown to be associated with increased incidence and severity of hypoglycaemia in people with diabetes [24, 25]. Furthermore, fifteen (27%) of the 56 participants had BMI < 24 kg/m^2^, the median among these was 22.8 kg/m2 (range 18.9–23.9). The BMI was beneath the recommended level (< 24 kg/m^2^) in one-third of the participants with one or more hypoglycaemicepisode, and 19% were at risk of being malnourished. In addition to being an independent risk factor for hypoglycaemic episodes [25], low BMI falsely gives a higher GFR when estimated by creatinine, hence the prevalence of decreased kidney function was most likely underreported.

The low median HbA1c identified in the study is in accordance with previous research showing that several older persons with diabetes may be overtreated with regard to glucose-lowering medication [3–6, 11]. Previous studies from nursing homes have shown that older individuals with diabetes using glucose-lowering medication are subjected to tighter glycaemic control than recommended in guidelines [3–6, 11]. Corresponding data for older home-dwelling people receiving home care services are unknown [1]. However, it has been shown that glycaemic control is too tight in nearly two-thirds of older people with diabetes and complex/intermediate or very complex/poor health [26], and that this most likely leads to more harm than benefits [27]. Choosing a treatment that can help reduce the risk of hypoglycaemia in older people is also essential. Using insulin analogues has been shown in RCTs to reduce the risk of hypoglycaemia [28], including in people with type 2 diabetes [29].

However, in our study, 5 of 16 participants using insulin analogues had at least one hypoglycaemic episode during the study period.

In our study, 86% of the participants who had a hypoglycaemic episode during the five-day period lived alone. Twenty of the participants did not recognize the symptoms of hypoglycaemia, and 40% of these had at least one hypoglycaemic episode during the study period. It is well known that older people with diabetes who live alone are at higher risk of more severe health decline due to poorer self-management [30]. Thus, this is an extra vulnerable group. Our findings emphasize the urgent need for health care services to consider how glucose monitoring routines for these participants can be improved. According to the recently published consensus report by the American Diabetes Association and the European Associations for the Study of Diabetes, it is essential to prescribe medication that avoids unnecessary harm, such as hypoglycaemia, in this population [30]. In addition, the Standards of Medical Care in Older People indicate that older people with diabetes with physical or cognitive limitations can benefit from more efficient use of new technology devices such as intermittent use of CGM [31]. This issue has already been elucidated in several studies [17, 22, 26, 27, 32–34], and our study adds further evidence to this suggestion. The use of CGM during the five-day assessment period in our study identified hypoglycaemic episodes and significant glucose fluctuations throughout the day, which would not have been identified without a CGM device.

Although many of the participants in our study had low HbA1c, there were also participants with an HbA1c > 64 mmol/mol (8%), who also had a hypoglycaemic episode during the study period. Overall, we found that one out of four participants with hypoglycaemia had a higher HbA1c than recommended. The fact that hypoglycaemia also may occur in those with high HbA1c levels has been shown in previous studies, as well [22, 25]. Accordingly, if diabetes therapy is evaluated based only on HbA1c, it might have disastrous consequences. Furthermore, HbA1c levels may be misleadingly high due to the combination of anaemia and use of erythropoietin [35]. It is known that the use of erythropoietin may result in falsely elevated HbA1c [36]. One of the participants with eGFR of 9 mL/min/1.73 m2 was using erythropoietin substitution but did not have a hypoglycaemic episode during the five-day period. In reviewing the patient information during the recruitment period, we tried to exclude those who were on medication that indicated severe kidney failure. Nevertheless, we saw that some people with severely reduced kidney function were included by the study nurses, being unaware at that time that they had severely reduced kidney function.

Using more specific information, such as information from intermittent CGM monitoring, can contribute to improved adaptation and individualization of treatment [27, 34]. Hence, CGM provides important and direct observation of glycaemic excursions and daily glucose variability, and thereby an overview of patterns of hypo- and hyperglycaemia. Previous studies have shown that CGM is an appropriate tool for monitoring asymptomatic hypoglycaemia [14, 37]. However, intermittent or continuous use of CGM in home care services is a question of costs, practical use, training, and organizational aspects that must be further addressed and debated.

Our study has strengths and limitations. We could not include all the eligible older people with diabetes for different reasons, and we did not include those with more severe cognitive dysfunction and severe comorbidities. Thus, generalizations should be made with caution. We cannot exclude that hypoglycaemia among home-dwelling older people with diabetes may be an even more or less widespread problem than reported in this study. Although all glucose levels below 3.9 mmol/L have been shown to have significant negative consequences, especially for older people, absolute danger occurs for most in the lower ranges below 3.0 mmol/L [33]. Using a blinded CGM device helped to ensure that the individuals with diabetes or the home care staff were not biased by the glucose values measured; we obtained an accurate picture of the actual situation among old home-dwelling people with diabetes. However, we cannot exclude that there are weaknesses related to the precision of CGM data. For example, pressure on the CGM sensor can result in too low or too high glucose levels. To avoid that, we attached the sensor to the stomach of the participants assuming that this group of older people rarely sleep in a prone position. Also, we cannot exclude that some of the participants may have used the over-the-counter drugs acetaminophen aspirin and vitamin C which can lead to falsely elevated glucose levels. Further, including a food diary might have strengthened the study. We collected such data in the pilot phase but experienced that the data collection was difficult and often incomplete. The relatively short study period of 5 days is also a limitation. The average time of measurement was slightly shorter than five days. One reason why there were some cut shorts was that switching on and off the sensor had to be done in a way that suited the study nurses in terms of their work schedule. In some cases, the CGM was connected in the afternoon and disconnected in the morning for practical reasons. Unfortunately, there were also a few cases where the CGM sensor loosened, stopped working, or was removed by the participant. Consensus guidelines recommend calculating CGM metrics using at least 70% of data from 14 days [32]. On the other hand, a recent study showed that CGM data from 7 days compared with 14 days did not show significant differences in glucose management indicators [38]. The results from the published feasibility study [39] showed that a five-day period was sufficient to produce relevant data. Moreover, the five-day study period allowed for conducting the CGM measures primarily on weekdays. In addition, a five-day study period was feasible in relation to the blinded glucose sensors that were available (the maximum duration of the IPro2 sensors is 6 days), and also the resources available for this study. We used an older version of the CGM sensor with a relatively higher MARD than the newer CGMs, but this was the only blinded version. Typically, a CGM system with a MARD < 10% is regarded as having good analytical performance [15]. However, no prospective clinical studies have evaluated the possible added clinical benefits of low MARD values [12, 14]. Nevertheless, when assessing hypoglycaemia using CGM, the accuracy of the data in the lower glycaemic range should be considered [14].

## Conclusion

This study identified an unacceptably high number of unknown hypoglycaemic episodes among older home-dwelling people with diabetes receiving home care, even among those not using insulin or sulfonylurea. The study indicates that hypoglycaemia may be far more common among this population than shown in earlier epidemiologic studies. The study provides essential knowledge that can serve as a foundation to improve the treatment and care for this vulnerable patient group. The routines for glucose monitoring and other prevention tasks need to be considered more comprehensively, also, among those treated with glucose-lowering medications other than insulin.

### Supplementary Information

**Additional file 1.** Clinical data for the total 56 participants with diabetes (≥65 years) receiving home care, and divided into subgroups of participants with no hypoglycaemic episode and participants with one or more hypoglycaemic episodes during the study period.

## Data Availability

Data are available from the corresponding author upon reasonable request.
