# Supplementary material for: High number of hypoglycaemic episodes identified by CGM among home-dwelling older people with diabetes: an observational study in Norway
Source: BMC Endocr Disord. 2023 Oct 10;23:218. doi: 10.1186/s12902-023-01472-6 (PMC10566065; doi:10.1186/s12902-023-01472-6)
Supplement: Supplementary file 1 — Additional file 1. Clinical data for the total 56 participants with diabetes (≥65 years) receiving home care, and divided into subgroups of participants with no hypoglycaemic episode and participants with one or more hypoglycaemic episodes during the study period. [file 12902_2023_1472_MOESM1_ESM.docx]

Clinical data for the total 56 participants with diabetes (≥65 years) receiving home care, and divided into subgroups of participants with no hypoglycaemic episode and participants with one or more hypoglycaemic episodes during the study period.

|  | Total | No hypoglycaemic episode | One or more hypoglycaemic episode |
| --- | --- | --- | --- |
| HbA1c (mmol/mol), median (range)* | 57 (34-108) | 60 (34-108) | 52 (38-80) |
| HbA1c (%), median (range)* | 7.4 (5.3-12.0) | 7.6 (5.3-12.0) | 6.9 (5.6-9.5) |
| eGFR (ml/min/1.73 m2), median (range)* | 69 (6-123) | 80 (9-123) | 61 (22-110) |
| Weight (kg), median (range)* | 78 (45-131) | 80 (45-131) | 75 (63-96) |
| Body mass index (kg/m2), median (range)* | 27 (19-41) | 28 (19-41) | 24 (21 -39) |

eGFR, estimated glomerular filtration rate.

*Missing data: HbA1c, n=3; eGFR (estimated glomerular filtration rate), n=3; weight, n=1; body mass index, n=2.
